# Supplementary material for: PEGylated surfaces for the study of DNA–protein interactions by atomic force microscopy
Source: Nanoscale. 2019 Sep 23;11(42):20072–80. doi: 10.1039/c9nr07104k (PMC6964798; doi:10.1039/c9nr07104k)
Supplement: Supplementary file 1 [file NR-011-C9NR07104K-s001.pdf]

Electronic Supplementary Material (ESI) for Nanoscale.
This journal is © The Royal Society of Chemistry 2019

PEGylated surfaces for the study of DNA-protein interactions by atomic force microscopy

Bernice Akpinar[a,b], Philip J. Haynes[a,b], Nicholas A. W. Bell[c], Katharina Brunner[c,d], Alice L.B. Pyne[a,e*], Bart W. Hoogenboom[a,f*]

[a] London Centre for Nanotechnology, University College London, 17-19 Gordon Street, London WC1H 0AH, United Kingdom

[b] Department of Chemistry, Imperial College London, SW7 2AZ, United Kingdom

[c] The Francis Crick Institute, 1 Midland Road, London, NW1 1AT, United Kingdom

[d] Discovery Biology, Discovery Sciences, R&D, AstraZeneca, 50F49, Mereside, Alderley Park, Macclesfield, Cheshire, SK10 4TG, United Kingdom

[e] Department of Materials Science and Engineering, University of Sheffield, S1 3JD, United Kingdom

[f] Department of Physics and Astronomy, University College London, Gower Street, London WC1E 6BT, United Kingdom

* E-mail: a.l.pyne@sheffield.ac.uk, b.hoogenboom@ucl.ac.uk

Supplementary Information

Notation as degree of polymerization	Notation as molecular weight / Da
$PLL_{1000\text{-}2000}$	$PLL_{150\text{-}300k}$
PLL_{10}-b-PEG_{22}	$PLL_{1.6k}$-b-PEG_{1k}
PLL_{10}-b-PEG_{113}	$PLL_{1.6k}$-b-PEG_{5k}
PLL_{100}-b-PEG_{113}	PLL_{16k}-b-PEG_{5k}
PLL_{10}-b-PEG_{454}	$PLL_{1.6k}$-b-PEG_{20k}

Supplementary table 1 | Table expressing molecular weights corresponding to degrees of polymerization for the reagents used in this study.

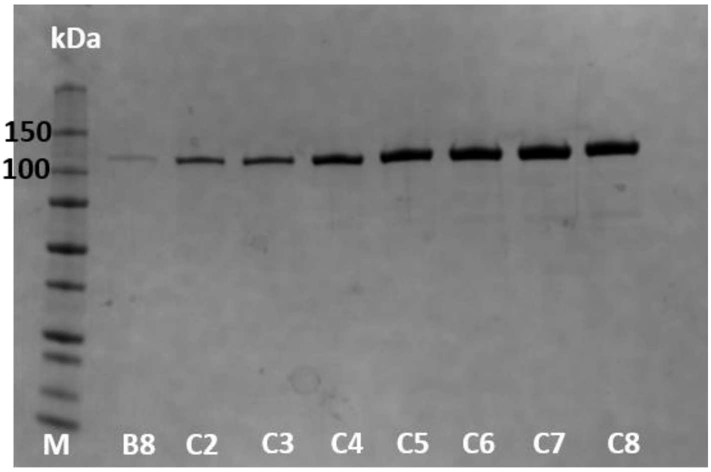

Supplementary figure 1 | PARP1 purification. SDS-PAGE gel of fractions eluted after size exclusion chromatography, as described in the Methods.

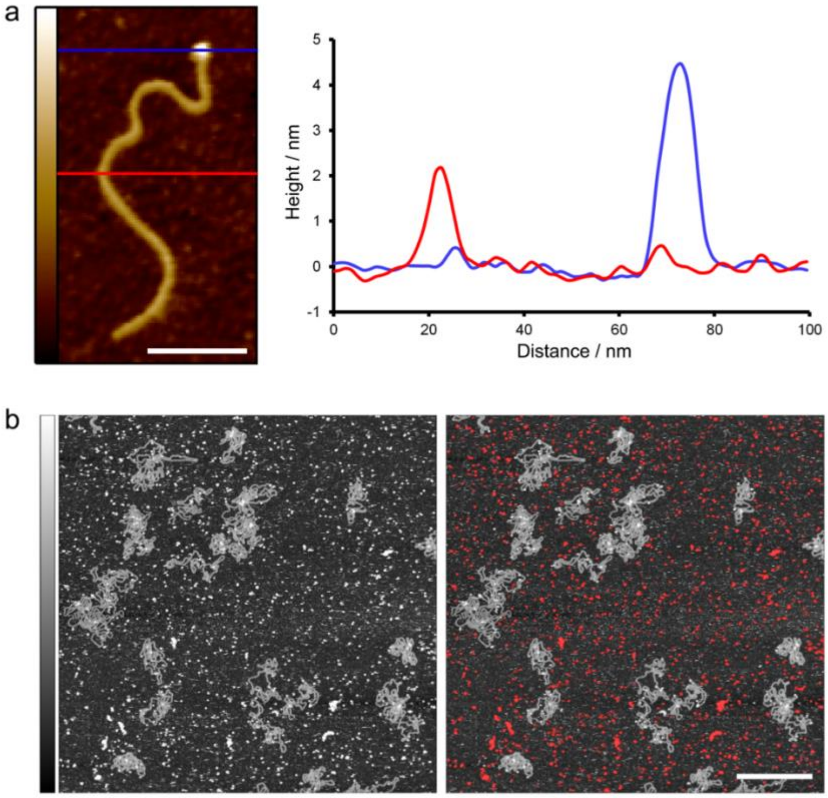

Supplementary figure 2 | Example height profile of DNA and Streptavidin along for masking data DNA height is around 2 nm, streptavidin around 4 nm from the background (a) Examples to show data is masked according to height and further filtered by feature size and height to calculate background coverage of streptavidin. Colour scale shown in (a) is 5 nm and shown in (b) 8 nm. Scale bar in (a) is 50 nm and in (b) 400 nm. All AFM images taken in solution.

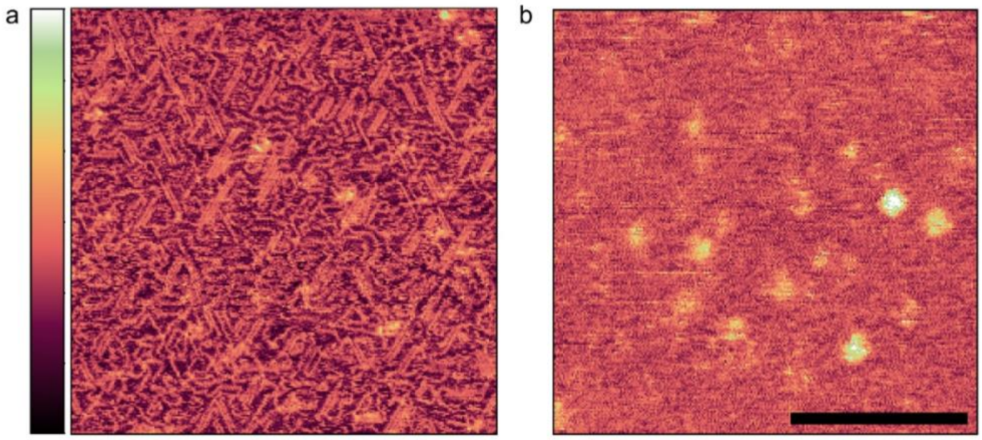

Supplementary figure 3 | AFM images of PLL-mica After deposition at low concentration of PLL, 0.001% solution incubated for 5 minutes before washing and imaging (a) and high concentration of PLL, 0.01% solution incubated for 1 minute before washing and imaging (b). Both images are taken in solution with sampling every 0.5 nm. Scale bar the same in both and is 50 nm. Height scale is shown on the left and is 3 nm.

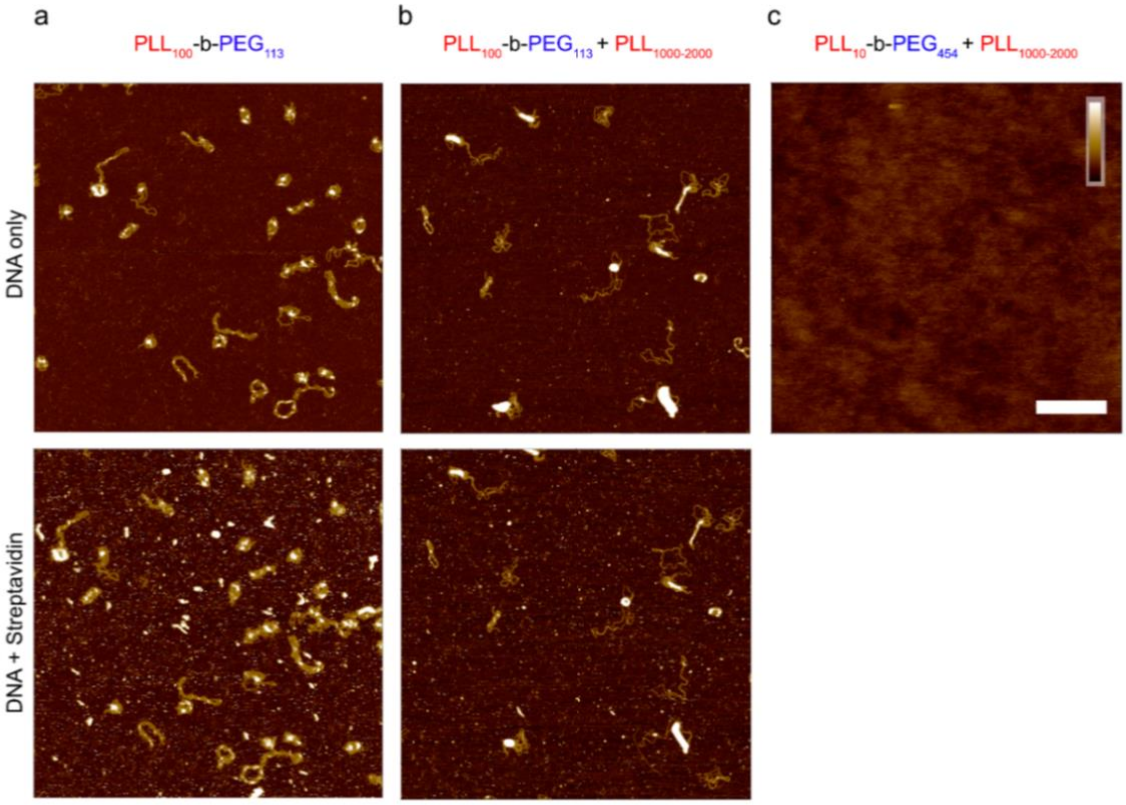

Supplementary figure 4 | Additional characterization of DNA plasmid and streptavidin adsorption on functionalized mica. Streptavidin (160 nM) was added after DNA immobilization on (a) PLL_{100}-b-PEG_{113} surface, (b) a mixed PLL_{100}-b-PEG_{113} and $PLL_{1000-2000}$ surface and (c) PLL_{100}-b-PEG_{454} surface. Inset colour scale 8 nm and scale bar 200 nm. Images taken in solution.

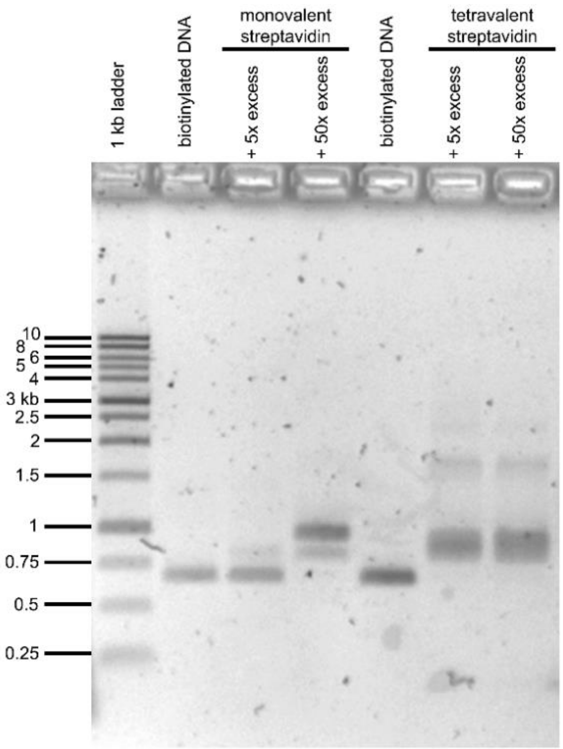

Supplementary figure 5 | Monovalent and tetravalent streptavidin binding to dual-biotin 672 bp DNA. Clear band shifts confirm binding of monovalent streptavidin when pre-incubated for 5 minutes at ~50x excess and tetravalent streptavidin when at ~5x excess over the number of biotin binding sites.

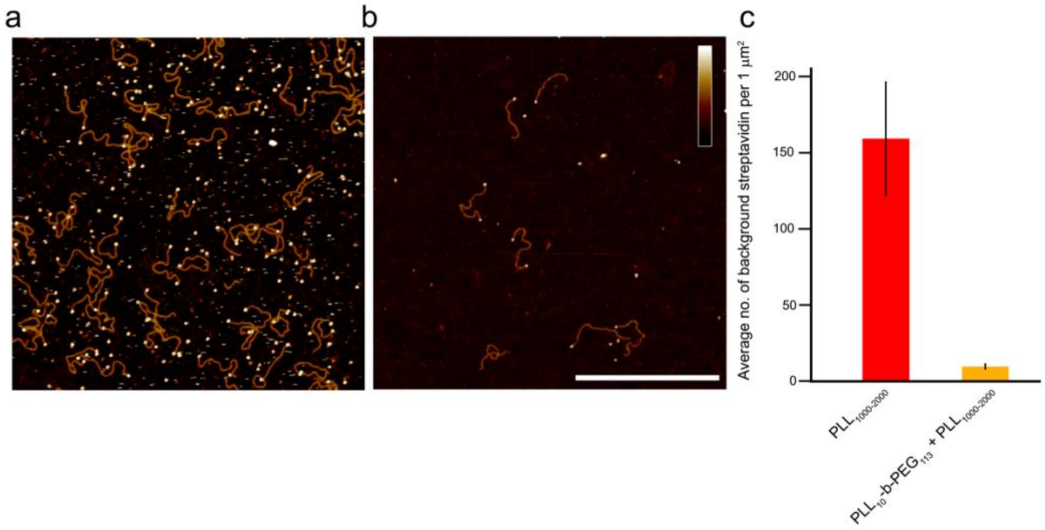

Supplementary figure 6 | Dual-biotin 672 bp DNA pre-incubated with monovalent streptavidin added to (a) $PLL_{1000\text{-}2000}$ and (b) PLL_{10}-b-PEG_{113} / $PLL_{1000\text{-}2000}$ surface. (c) a graph of the average number of streptavidin molecules bound to the background (i.e. not on the end of a DNA molecule) per 1 μm^2 analyzed for passivated surface (mean ± standard deviation, n = 8 images, 32 μm^2) and for the PLL surface (n = 7 images, 28 μm^2). Scale bar is 400 nm and height scale is 6 nm. AFM images taken in solution.
